# Blockchain for Vehicular Internet of Things: Recent Advances and Open Issues

**DOI:** 10.3390/s20185079

**Published:** 2020-09-07

**Authors:** Chunrong Peng, Celimuge Wu, Liming Gao, Jiefang Zhang, Kok-Lim Alvin Yau, Yusheng Ji

**Affiliations:** 1 Inner Mongolia University of Finance and Economics Library, Inner Mongolia University of Finance and Economics, Hohhot 010051, China; pengchunrong1978@gmail.com; 2 Graduate School of Informatics and Engineering, The University of Electro-Communications, Tokyo 1828585, Japan; gaolm0660@comp.is.uec.ac.jp; 3 Institute of Intelligent Media Technology, Communication University of Zhejiang, Hangzhou 310018, China; 20100937@cuz.edu.cn; 4 School of Science and Technology, Sunway University, Petaling Jaya 47500, Selangor, Malaysia; koklimy@sunway.edu.my; 5 Information Systems Architecture Research Division, National Institute of Informatics, Tokyo 1018430, Japan; kei@nii.ac.jp

**Keywords:** blockchain, vehicular networks, IoT, decentralization

## Abstract

The vehicular Internet of Things (IoT) comprises enabling technologies for a large number of important applications including collaborative autonomous driving and advanced transportation systems. Due to the mobility of vehicles, strict application requirements, and limited communication resources, the conventional centralized control fails to provide sufficient quality of service for connected vehicles, so a decentralized approach is required in the vicinity to satisfy the requirements of delay-sensitive and mission-critical applications. A decentralized system is also more resistant to the single point of failure problem and malicious attacks. Blockchain technology has been attracting great interest due to its capability of achieving a decentralized, transparent, and tamper-resistant system. There are many studies focusing on the use of blockchain in managing data and transactions in vehicular environments. However, the application of blockchain in vehicular environments also faces some technical challenges. In this paper, we first explain the fundamentals of blockchain and vehicular IoT. Then, we conduct a literature review on the existing research efforts of the blockchain for vehicular IoT by discussing the research problems and technical issues. After that, we point out some future research issues considering the characteristics of both blockchain and vehicular IoT.

## 1. Introduction

The dynamic feature and resource limitation of vehicular environments have posed significant challenges to the design of an efficient vehicular IoT system [1,2]. The conventional cloud computing framework requires the transmission of vehicle data to the cloud, resulting in a high latency. To solve this problem, the mobile edge computing (MEC) technologies in vehicular networks have attracted a high degree of attention [3]. Future vehicular IoT systems must cater to the unprecedented high reliability and ultra-low latency requirements in order to enable emerging applications, including collaborative autonomous driving and intelligent control of traffic signals. The application requirements also vary with application types, time, location, and other contexts, including node density, vehicle velocity, and so forth. The complex and dynamic features of vehicular environments make the problem more challenging since a system perfectly working on the current road scenario may fail in different scenarios. This requires an intelligent solution that optimizes one’s own behaviors according to the changes of the environment in an online manner [4].

Existing solutions for vehicular IoT applications mainly focus on the intelligence of each vehicle and do not address collaboration among vehicles. For example, each autonomous driving car aims to optimize its own actions based on its own sensors’ readings, which may not efficiently utilize knowledge perceived by other vehicles, resulting in latency in adapting to the changes in the environments. Since the environment (e.g., area) that a single vehicle can observe is always limited, it is difficult to achieve a satisfactory outcome in a dynamic environment. Vehicle-to-vehicle (V2V) and vehicle-to-everything (V2X) communications have been introduced to provide a way of information exchange between different entities in vehicular networks. Based on these communications, the coordination among different entities, including vehicles, roadside units (RSUs), base stations, pedestrians, and so forth, can be achieved. However, it is difficult to achieve an efficient collaboration among multiple entities that belong to different owners due to privacy and management issues.

Federated learning [5,6], also known as collaborative learning, is a distributed learning technology that enables knowledge sharing between different vehicles with privacy protection. In federated learning, each vehicle (client) trains a local model based on the sensor data it perceives and uploads the trained model to the central server. The central server then aggregates local models uploaded by different vehicles and generates a global model. By sharing the global model with all the vehicles, each vehicle is able to utilize the knowledge of other vehicles without sacrificing privacy. Depending on the central server, federated learning is not applicable for most scenarios in vehicular networks due to limited network resources and latency concerns. Therefore, collaboration in vehicular networks should be “decentralized” in nature.

**Figure 1:**
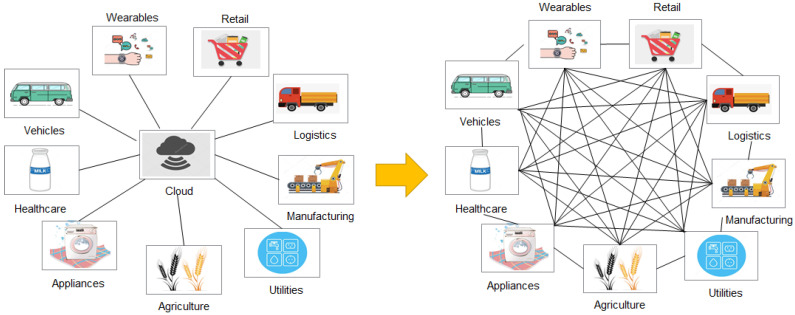
Decentralization of management using blockchain.

With the development of cryptography, consensus algorithms, game theory, distributed systems, and communication technologies, a new distributed ledger technology (DLT) called blockchain was introduced by Satoshi Nakamoto in 2008 for supporting bitcoin, a well-known cryptocurrency [7]. As shown in Figure 1, the emergence of the blockchain technology has brought a new way of thinking about how to solve the painful points of the current IoT systems [8,9]. Blockchain is a technology that links transaction blocks and a list of records using cryptography to maintain transaction histories in a decentralized manner. In [10], blockchain is defined as a “digital, decentralized and distributed ledger in which transactions are logged and added in chronological order with the goal of creating permanent and tamperproof records”. Blockchain is based on an open ledger that provides a verifiable and permanent way to manage transactions in a decentralized environment, and it has inspired many researchers to investigate the use of blockchain in vehicular IoT. Blockchain has the following key advantages:

- Decentralization: Blockchain enables a distributed system without a central controller. By using the cryptography technology to prove the relationship between two adjacent blocks, blockchain can show the global consensus without the need of a validated third-party.
- Irrevocability and traceability: Since the relationship between two adjacent blocks is certified by hash functions, the change of a single bit in a block can cause inconsistency in the rest of the blocks, resulting in the irrevocability of the blockchain. This feature enables immutable records of all the transactions. By disclosing all the blocks to all the participants, blockchain is able to provide traceability to all the agreements made by the public.
- Fault tolerance: Blockchain can achieve a global consensus without the need for a centralized authority. This enables a better tolerance to node faults, such as when under a security attack or during a disaster. This feature can be used to improve the security level of a system or to design a fault-tolerant system.

These features have facilitated the rapid expansion of the use of the blockchain technology in various sectors, including payments, finance, supply chain, healthcare, insurance, asset management, and so forth. Many vehicular IoT systems require vehicles to conduct fast and accurate actions in decentralized environments. However, there are some important issues that must be further investigated in order to make blockchain applicable in vehicular environments:

- Decentralized consensus with imperfect information: Blockchain enables a distributed system without a central controller. A critical problem is how to achieve a consensus in a complex vehicular environment where each node has only limited and imperfect information.
- Effect of vehicle mobility: The mobility of vehicles poses challenges to the management of blockchain. A critical problem is how to achieve a consensus in a frequently changing environment without sacrificing the consistency of a distributed system.
- Effect of consensus delay: Most vehicular IoT applications require a low latency, which poses a great challenge to the design of a blockchain that incurs a certain amount of time before reaching a consensus. Therefore, it becomes particularly important to design a consensus algorithm that is reliable and lightweight.
- Dissemination of blocks: Blockchain needs to disseminate blocks to the whole network in order to reach an agreement. Since the wireless resource in the vehicular networks is limited, the efficiency of the dissemination of blocks directly affects the performance of the system. Therefore, it is urgent to address the problem of how to ensure that the ledgers can be distributed to all nodes in the network efficiently.

With features like seamless authentication, distributed data storage, transparency, and anonymity, the blockchain technology and vehicular IoT have a mutual attraction. This paper presents the basic mechanism and a holistic classification of blockchain, as well as the reason why blockchain and vehicular IoT are essential to each other. For the purpose of pointing out the technical challenges of applying blockchain in the vehicular IoT scenarios, this paper first introduces the fundamentals of the blockchain technology and then conducts a survey on the related studies. Then, the future research directions on the use of blockchain in vehicular IoT are discussed by considering the characteristics of vehicular environments and the blockchain technologies. The main contributions of this paper are as follows:

- We present a review of the frameworks and recent advances of blockchain technologies in vehicular IoT environments. To the best of our knowledge, this is the first paper of its kind.
- We discuss the technical issues when applying blockchain technologies in vehicular IoT from two perspectives. First, we discuss the characteristics of vehicular IoT environments and the corresponding requirements on the blockchain performance by explaining potential enhancements to existing studies, including vehicular IoT protocols, and give some discussions on how to support blockchain applications by designing efficient vehicular IoT protocols.
- We point out future research directions regarding the use of blockchain in vehicular IoT and explain some concepts and possible ways to promote related studies.

Since there is no room for including all the related studies in the paper, considering the tradeoff between the number of references and the efforts required for understanding the paper, we select research papers based on the relevance to the topic, the quality, the publication venue, the publication date, and the number of citations. We put special focus on the papers that have been published by IEEE journals/magazines in the past three years. We also include the papers that receive a high number of citations.

The rest of the paper is organized as follows. We first describe the fundamentals of blockchain, including definitions, basic features, classifications, and application scenarios in Section 2. Section 3 explores the technical issues and existing studies on the use of blockchain in solving vehicular IoT problems. Section 4 discusses the characteristics and limitations of the existing studies. Section 5 points out future research directions from the perspectives of both vehicular IoT and blockchain technologies, and finally, Section 6 draws our conclusions.

## 2. Blockchain Fundamentals

### 2.1. Main Concept

At present, there is no widely recognized definition for blockchain. Unlike most new-generation information technologies (e.g., cloud computing, big data, and IoT) that can function independently on their own accord, blockchain technology is a combination of existing technologies to achieve some objective functions. Broadly speaking, blockchain technology is a new distributed infrastructure and computing paradigm that uses a special pattern of structure to verify and store data, distributed consensus algorithms to generate and update data, cryptography to ensure the security of data transmission and access, as well as smart contracts comprised of automated script codes to program and manipulate data. In a narrow sense, blockchain is a cryptographically guaranteed, tamper-resistant, and unforgeable distributed ledger (or a chained data structure) that sequentially combines data blocks in a chronological order. The rest of this section presents the five main characteristics of blockchain, namely, decentralization, transparency, autonomy, tamper-resistance, and anonymity.

“Decentralization” means that blockchain is composed of many nodes to form a peer-to-peer network. There is no centralized equipment and management organization. The verification, accounting, storage, maintenance, and transmission of blockchain data are implemented using mathematical algorithms, rather than based on central institutions. “Decentralization” enables nodes in the network to connect freely and exchange data, assets, and information. Note that, “distributed” is different from “decentralized”. Some “distributed” systems may be centralized. For example, a central server distributes data to multiple servers to enable distributed parallel processing of the data. However, in a decentralized system, there is no such kind of central server.

“Transparency” means that all data information of the blockchain is public, whereby each transaction is broadcast and is visible to other nodes. The consensus mechanism and rules set in the blockchain network can be verified by consistent and open-source source codes. Anyone can join the blockchain either freely or in a permissioned way for the case of permissioned blockchain systems.

“Autonomy” means that anyone can participate in the blockchain network and each node can get a complete copy of the database. Nodes maintain a common blockchain through competitive computing based on a set of consensus mechanisms. Blockchain technology uses consensus-based specifications and protocols to enable all nodes in the entire system to exchange data freely and securely in a trusted environment, in which any human intervention does not work.

“Tamper-resistance” means that the modification of the database by a single or even multiple nodes cannot affect the database of other nodes unless more than 51% of the nodes are controlled at the same time to modify the database. The blockchain uses a hash function and an asymmetric encryption mechanism of the cryptography technique to ensure no tampering with the information of the blockchain. Since each block is linked with the previous block by a cryptographic proof, the block must be modified to change the transaction content in a historical block when the blockchain reaches a certain length. The transaction records and cryptographic proofs of all previous blocks are reconstructed, effectively preventing tampering.

Since the exchange of information between nodes follows a fixed algorithm based on a decentralized consensus, the real identity can be protected, resulting in “anonymity”. The program rules in the blockchain determine whether an activity is valid or not. Therefore, the counterparty does not need to disclose the real identity.

The biggest advantage of the blockchain technology is decentralization. Blockchain integrates cryptography, the consensus mechanism, and other techniques to enable peer-to-peer transactions in decentralized environments. Therefore, blockchain has become one of the key underlying technologies for digital currency systems [11].

### 2.2. Development Roadmap of Blockchain

In 2009, a scholar or organization named Satoshi Nakamoto published the Bitcoin white paper entitled “A Peer-to-Peer Electronic Cash System” [12], which is considered as the origin of blockchain technology. With the increasing popularity of digital currencies such as Bitcoin, the development of blockchain technology has attracted widespread attention from government agencies, financial institutions, and research institutions. The research and application results of blockchain have shown a trend of a geometric progression and are closely integrated with big data, IoT, intelligent manufacturing, and other scenarios. Regarding the development of the blockchain technology, the blockchain evolution has gone through two stages:

- A blockchain model featuring a programmable digital cryptocurrency system, represented by Bitcoin.
- A blockchain model with the programmable smart contract as the main feature, represented by Ethereum [13].

However, the blockchain model has experienced a parallel development rather than a qualitative evolution. In other words, the two stages of the blockchain model currently exist at the same time, and the first stage, being represented by digital cryptocurrency, is still under exploration. The different stages of the development of blockchain present mutual influences that complement each other.

### 2.3. Classification of Blockchain

**Figure 2:**
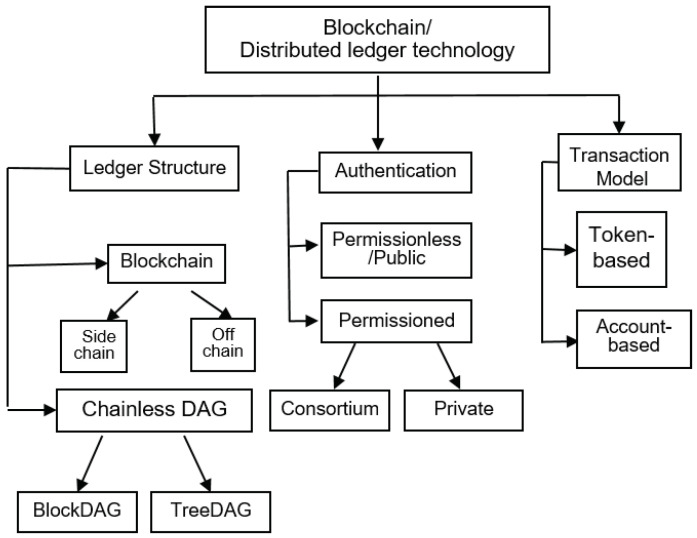
Classification of blockchain.

As shown in Figure 2, there are different types of blockchain frameworks depending on the types of managed data, the availability of such data, and the actions that can be performed by a user. We can classify the existing blockchain frameworks into different categories from different perspectives, including “authentication”, “ledger structure”, and “transaction model”.

#### 2.3.1. Authentication

Some authors or papers use the public/permissionless and private/permissioned terms as synonyms when referring to cryptocurrencies, but they are different from the IoT point of view where it is important to distinguish between authentication (accessibility; private versus public) and authorization (capability; permissionless versus permissioned) [14]. In terms of the permissioned/public chain, the difference is not significant. However, in the permissioned chain, besides the private chain, there is another division called the consortium chain. Nonetheless, such distinctions are still in debate. A comparison of different types of blockchain frameworks is shown in Table 1, and the characteristics of each type are shown below.

- Permissionless chain (public chain): In the public chain, any individual or group can send a transaction, which can obtain a valid confirmation of the blockchain. Everyone can participate in the consensus process. The public chain, such as Bitcoin or Ethereum, is the earliest and the most widely used blockchain at present.
- Permissioned chain: Each node participating in this kind of blockchain system must first get a permission. Nodes without permission are not accessible to the system. Therefore, both the private chain and the consortium chain belong to the permissioned chain category.
  - Consortium chain: Multiple accounting nodes are specified within a group, and the generation of each block is determined by all pre-selected nodes. The pre-selected nodes participate in the consensus process, and other access nodes can participate in the transaction.
  - Private chain: Using only blockchain general ledger technology for accounting and providing exclusive access to an organization or individual.

#### 2.3.2. Ledger Structure

The ledger structure reflects the data structure type used in the blockchain technology, which is used as an immutable database for transactions. The blockchain data structure falls into two categories: chain structured and directed acyclic graph (DAG) structured. They can be further divided into subcategories as shown in Figure 2. The chain structure includes sidechain and off-chain, while chain-free DAGs are comprised of BlockDAG and TreeDAG.

- Chain structure: The chain structure contains a set of trustless participating nodes (devices) that share a common ledger database without the involvement of middlemen. In a traditional blockchain like Bitcoin, blocks are generated by the miners that have solved a complex cryptographic puzzle. Each block must refer to the previous block, so the longest chain is also the most difficult to overthrow and tamper with. The nodes always think that the longest chain is the effective blockchain, and only miners who mine on the longest chain can get rewards, which also helps to guide the blockchain system to reach a universal consensus and prevent malicious acts such as double-spending. In addition to the classic blockchain data structure, there are some newly introduced chain structures as follows.
  - Sidechain: The sidechain is a protocol that permits developers to connect new sidechains to a blockchain that is already in operation (e.g., Bitcoin). These sidechains with different properties can implement transactions between the main chain and sidechains and fulfill various demands in different scenarios. However, the sidechain usually does not possess a comparable computing capability to the main chain, so it is more vulnerable than the main chain.
  - Off-chain: The off-chain structure provides a transfer mechanism that happens outside the main chain. When a transaction is executed on the off-chain, it is only registered on a local ledger and is synchronized to the main chain periodically or on demand. The off-chain structure has a much higher throughput than the main chain, but at the same time, the off-chain transaction is risky since it is hard to confirm the validity of the transaction.
- Chainless structure: Low throughput in the traditional blockchain limits the development of blockchain technology. The first issue is the scalability of blockchain. When the transactions on blockchain are frequent, the performance of blockchain declines linearly. To overcome this bottleneck of the blockchain systems, a different blockchain data structure has been designed to employ a DAG structure to store the data.
  - BlockDAG: In a chain-based structure, only the longest chain is valuable. Therefore, only one miner conducts effective work, and all the other efforts are wasted. To overcome this drawback of the blockchain system, a blockDAGstructure is proposed. It comprises blocks in a DAG. Blocks may refer to multiple predecessors instead of a single parent; therefore, they transform the consensus progress from being serial to being parallel. Usually, the blockDAG system conducts the consensus via ordering instead of the longest chain rule.
  - TreeDAG: Unlike the blockchain structures mentioned above, a TreeDAG does not have a block-based structure, and it is based on every single transaction that contains hashes from previous transactions to form a tree-like directed acyclic graph (TDAG). The transactions in DAG can be regarded as “blocks”, but these blocks can also be used as nodes to form a complex network topology. Each node can be a trader and a verifier at the same time because the transaction processing in the DAG is done by the transaction node itself. This is so that the more transactions and more nodes in the network, the faster the processing will be, which naturally matches the characteristics of IoT.

#### 2.3.3. Transaction Model

The type of blockchain can also be divided into two transaction models, namely the token-based model and the account-based model. These two types of models also correspond to the two development stages of blockchain.

- Token-based model: This type of blockchain model employs the token system, and it is usually used for tracking digital assets, such as Bitcoin, which corresponds to the first stage of the blockchain development.
- Account-based model: This type of blockchain, which may employ the token system, runs certain logics (e.g., smart contracts) on the ledger structure. The smart contract is an account-based script running on the ledger designed to disseminate, verify, or enforce contracts in an informational manner, thus enabling trusted transactions without third parties [15].

### 2.4. Blockchain Use Cases in the Era of IoT

**Figure 3:**
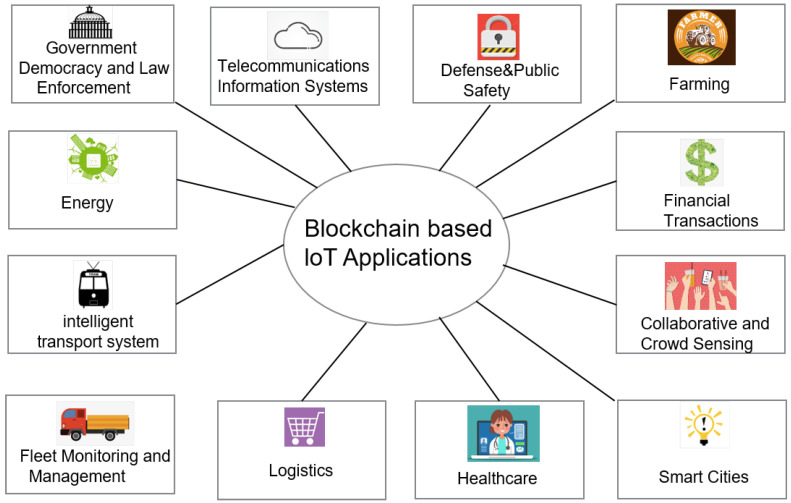
Blockchain-based IoT applications.

The blockchain system has a wide range of features like distributed high-redundancy storage, time-series data, tamper and counterfeit resistance, decentralized trust, automated execution of smart contracts, and security and privacy protection. Therefore, blockchain can be applied not only to the field of digital cryptocurrency [16], but also to various IoT applications, as shown in Figure 3. This section focuses on two main applications, namely supply chain management and healthcare management.

#### 2.4.1. Supply Chain Management

The combination of blockchain and IoT technologies is one of the most important applications of the blockchain technology that may bring revolutionary changes to the field of supply chain management. Traditional supply chain management faces problems such as inefficiency and coordination difficulties, due to information asymmetry, particularly in process tracking and overall arrangement. The blockchain technology enables open and transparent trading network information, which can greatly reduce information asymmetry and improve the efficiency of supply chain turnover. At the same time, the blockchain data cannot be falsified, and the traceability of transactions can effectively curb the problem of counterfeit and shoddy products in supply chain management [17].

#### 2.4.2. Healthcare Management

**Figure 4:**
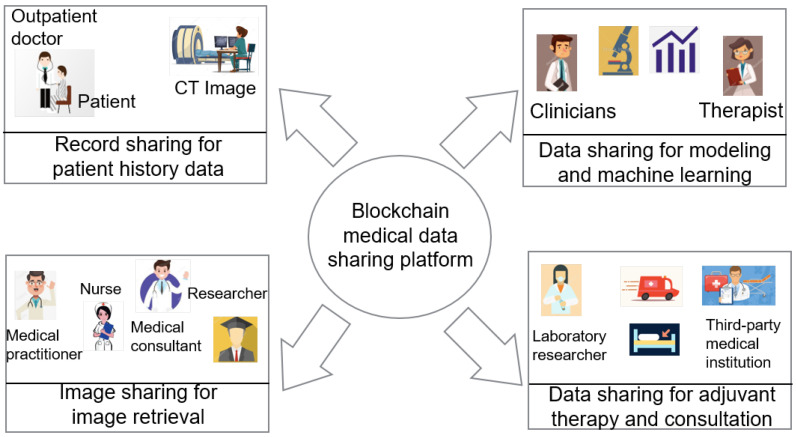
Potential applications of blockchain in the healthcare field.

The combination of blockchain and healthcare is one of the hotspots of current blockchain studies. The pain point of medical data sharing mainly lies in the privacy protection of sensitive patient information and the secured sharing of data by multiple organizations. As a multi-party maintenance, full back up, information security distributed data storage and sharing technology, Blockchain is a potential breakthrough for medical data sharing. Figure 4 shows the potential applications of blockchain in the healthcare field [18].

From the privacy protection point of view, the transmitted medical data are encrypted and stored securely in the blockchain, making it difficult to tamper with. All user information is anonymous, and thus, it is difficult to trace the data source without permission. In terms of the data form and content, it can be changed according to the needs of the data sharing type. For example, for an image, the features of the image can be stored as encrypted data.

From the access control point of view, most challenging problems in healthcare and medical treatment areas, such as the complexity of the medical insurance process, the difficulty of the settlement, the barriers to access between various medical institutions, and the problem of non-circulation of information, can be solved by building blockchain-based medical devices and treatment data platforms.

#### 2.4.3. Other Aspects of Blockchain-Based IoT Applications

Both blockchain and IoT are distributed peer-to-peer (P2P) networks. Therefore, the blockchain technology can be applied in almost all IoT scenarios.

- Product traceability: The tamper resistance, distributed storage, and other characteristics of blockchain technology provide solutions to the lack of trust in the traceability industry. Blockchain provides a transparent mechanism for information flow, logistics, and capital flow.
- Energy treading: Blockchain can help to implement retail energy trading for renewable energy and microgrid energy trading locally at a very low cost, which is difficult for electricity companies [19].
- Verification of digital identity: Blockchain can solve data sovereignty and privacy issues in digital identity without individual information disclosure.

#### 2.4.4. Issues and Challenges

The application of blockchain in IoT fields is still in its infancy. Although the blockchain technology combines multiple technologies such as cryptography and distributed storage, this does not mean that it has no loopholes. The blockchain system must involve a large number of nodes to prevent 51% attacks, selfish mining, and other types of attacks [20]. If the number of nodes participating in the calculation is too small, it is prone to 51% attacks, which can seriously threaten the operation and value of the network system. Another example is the private key and terminal security. In the current blockchain mechanism, the private key is stored locally in the user terminal. If the user’s private key is stolen, it can cause serious damage to the user’s digital assets.

Moreover, the security of consensus mechanism is an important aspect that needs much attention. At present, besides proof-of-work (PoW), many other consensus mechanisms, such as proof-of-stake (PoS) and delegated proof-of-stake (DPoS), have been proposed; however, whether they can achieve security and credibility satisfactorily has not been fully proven [21]. Besides, it is possible to cause network congestion through traditional cyber attacks, such as forcing a hard fork in the blockchain network, which leads to doubts about the credibility of the entire blockchain system. Finally, the drafting of technical standards and legal norms must match the development pace of blockchain [22,23]. In the rest of the paper, we discuss these issues by considering the characteristics of vehicular IoT.

## 3. Blockchain and the Vehicular Internet of Things

Here, we first explain the open technical issues of vehicular IoT and related studies. Then, we review the existing efforts of using blockchain in vehicular IoT environments by classifying existing studies into different categories based on the problems that blockchain technologies solve.

### 3.1. Vehicular IoT Layers

There are many studies discussing the technical challenges of vehicular IoT. Here, we show a brief summary of these studies according to the layers of IoT, namely, the perception layer, networking layer, and application layer.

#### 3.1.1. Perception Layer

The main function of the perception layer is to perceive the environments by using different types of sensor technologies, including the global positioning system (GPS), laser imaging detection and ranging (LiDAR), cameras, and so forth. Delay-sensitive or mission-critical vehicular IoT applications require a high-accuracy positioning approach in the control of vehicle behaviors. Since the conventional positioning systems, such as GPS, are unable to provide a satisfactory result, some studies discuss how to improve the positioning accuracy. In [24], Jo et al. proposed an approach to improve the accuracy of GPS by using the in-vehicle sensor information. Soatti et al. [25] discussed the use of information sharing between vehicles to improve the positioning accuracy at each single vehicle, which is called cooperative positioning. The sharable information includes traffic lights and some static objects, such as inactive cars in the surrounding areas. Shieh et al. [26] proposed an approach to use relative positions of vehicles calculated by measuring the coming directions of wireless signals based on two one-dimensional signal-direction discriminators. Williams and Barth [27] discussed the application requirements of vehicle positioning in vehicular environments.

Some studies discuss how to use compressed video sensing (CVS) technologies in the perception. In [28], Guo et al. employed a convolutional neural network (CNN)-based approach to improve the perception accuracy by analyzing the temporal correlation between video frames. Alasmary et al. [29] discussed the use of roadside camera sensors in vehicle sensing. They discussed the relationship between the number of sensors and vehicle mobility level.

The cooperative perception is an advanced way to improve the perception accuracy by facilitating the collaboration among multiple vehicle sensors. In [30], Ding et al. discussed the use of kinematic information in the cooperative perception for the purpose of meeting the reliability and delay requirements. Huang et al. [31] proposed a probabilistic approach to select sensor data in order to reduce the overhead for the cooperation without losing the accuracy. They proposed p-consistence, a data selection scheme, to make a tradeoff between communication overhead and system reliability. The scheme allows sensor vehicles to change the transmission frequency of the sensor data based on the vehicle density, sensor penetration rate, and road topology. Simulation results show that the p-consistence scheme can reduce the communication overhead while achieving an acceptable perception result.

#### 3.1.2. Networking Layer

The main objective of the networking layer is to provide efficient data transfer between different IoT entities. Multiple types of communication approaches are available for vehicular IoT, including cellular communications, IEEE 802.11p, and mmWave. As the international default standard for V2V communications, IEEE 802.11p is mainly used to supplement cellular communications especially for some scenarios where the cellular communications are unavailable or incurring higher latencies is possible. The vehicle mobility, limited transmission range, and various vehicle densities pose challenges to the design of networking protocols. V2V multi-hop communication protocols are discussed in vehicular environments for both connected scenarios [32,33] and delay-tolerant networks (DTN) [34,35].

V2X communications can be classified into two different categories, specifically unicast communications and broadcast communications, according to the number of receivers for each transmission. While the aforementioned studies [32,33,34,35] discussed unicast communications, the broadcast communications are used to disseminate control messages and safety-related messages. Since it is difficult, if not impossible, to conduct efficient retransmissions of broadcast frames at the MAC layer, the reliability of broadcast communications becomes more difficult to achieve as compared to unicast communications [36,37,38]. The broadcast protocols should take into account both reliability and efficiency since inefficient broadcasting can cause the broadcast storm problem [39,40].

Some studies discuss the resource allocation problem in vehicular IoT environments. In [41], the transmission scheduling problem in cognitive vehicular environment was discussed. The importance of handling an efficient handover in V2X communications was discussed in [42]. The radio resource allocation was optimized by deep reinforcement learning in [43].

#### 3.1.3. Application Layer

The studies related to the application layer issues cover computation offloading [44,45], task migration [46], and application frameworks [47,48,49,50]. In [44], an edge computing concept based on autonomous vehicles was proposed. Wang et al. [45] proposed an approach that uses game theory to improve the computation offloading process. Zhang et al. [46] discussed the technical issues in task migration between different entities by considering the offloading delay and formulated the task migration problem as a finite horizon Markov decision process. New vehicular IoT applications were proposed in [47,48,49,50]. In [47], a vehicular IoT platform for car parking was discussed. A cooperative and decentralized data management scheme was proposed in [48]. Khattak [49] discussed the use of vehicular IoT technologies in smart city applications. More emerging vehicular IoT applications can be found in [50].

### 3.2. Blockchain and Vehicular IoT

Blockchain has been attracting increasing interest in the past few years. There are some survey papers reviewing the existing studies regarding the use of blockchain in IoT environment. Ali et al. [51] reviewed the application of blockchains in IoT focusing on blockchain-based platforms, applications, and services. Dai et al. [52] focused on blockchain for fifth-generation (5G) and beyond networks for IoT. Ferrag et al. [53] surveyed existing blockchain protocols designed for IoT networks by considering different application domains, such as the Internet of Vehicles, Internet of Energy, Internet of Cloud, and edge computing. Viriyasitavat et al. [54] focused on existing studies in adopting blockchain to improve the security level of IoT applications. Similarly, the existing blockchain solutions targeting the security enhancement of IoT were surveyed by Alotaibi et al. [55]. Yang et al. [56] conducted a survey on blockchain-based frameworks from the perspective of Internet services.

**Figure 5:**
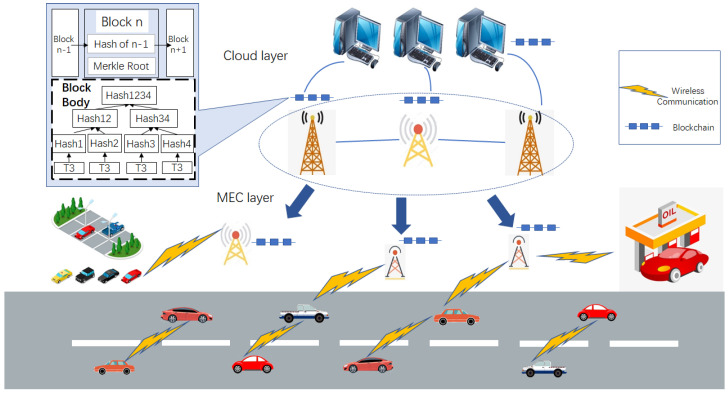
Blockchain in vehicular IoT.

Blockchain can be used to enable different types of interesting applications, including parking space management, traffic safety, and so forth, by enabling decentralized management of systems (see Figure 5). However, a comprehensive survey on the use of blockchain in vehicular IoT environments is still absent. Due to the vehicle mobility, various node densities, and strict QoS requirements of emergency applications, the vehicular IoT shows totally different features. Thus, existing blockchain technologies targeted for typical IoT environments cannot satisfy the requirements of vehicular IoT. This incurs the need to conduct a thorough study of the existing studies and technical challenges of the integration of blockchain and vehicular IoT. In this section, we give an overview of recent studies on the use of blockchain technologies for vehicular IoT by classifying these works into three different categories based on the IoT layered structure, namely the perception layer, networking layer, and application layer.

### 3.3. Perception Layer

Conducting an efficient perception in a vehicular environment is a challenging task because of the following reasons. First, the complex vehicular environment makes the use of simple sensing technology insufficient, requiring a cooperative sensing technology to achieve a high perception accuracy. Second, the vehicle mobility and decentralized feature of vehicular networks have increased the difficulty of judging the trustworthiness of sensor information acquired from other vehicles. Therefore, existing blockchain approaches mainly focus on solving the trust management problem of vehicles in decentralized vehicular networks, as shown in Table 2.

Li et al. [57] discussed the positioning accuracy in cooperative positioning scenarios where attackers or selfish nodes exist. They used deep neural networks to estimate positioning errors. They utilized landmark objects that have accurate position information to calculate the positioning errors and then trained the neural networks based on these data. When there is no landmark objects, the positioning errors are predicted using the speed, acceleration, driving direction, and GPS position information of vehicles. They used blockchain technology to store and share the evolution of positioning errors in order to protect the security of cooperative vehicles. The consortium blockchain technology is used instead of the public blockchain considering the processing speed and the dynamic feature of vehicular environments. The smart contract technology is integrated to automate the recording of positioning errors and improve the positioning accuracy. The mobile edge computing nodes, such as RSUs, are used as miners to maintain the blockchain and achieve the consensus.

Yang et al. [58] proposed a blockchain-based decentralized trust management system where RSUs work as miners to create blocks. The trust evaluation has the following steps. First, each vehicle collects messages sent by different vehicles and then uses the Bayesian inference model to calculate the credibility of a message *m*. The distance between the message sender and the event location is considered in this calculation based on the rationale that “vehicles closer to the event location are usually easier to conduct more accurate evaluation of the event”. After performing a Bayesian inference-based evaluation, each vehicle sends $\left(vehicle_{dst},vehicle_{src},messge_{id},rating\right)$ to nearby RSUs where $vehicle_{dst}$ and $vehicle_{src}$ are the receiver vehicle ID and sender vehicle ID of message $messge_{id}$, respectively. The trust value for the message, rating, is a numerical value raging between −1 and one. The rating of the messages is uploaded periodically to the RSUs, and the RSUs calculate the trust value of each vehicle by aggregating reports from all the vehicles. The RSUs then add the pair of vehicle ID and the corresponding trust value of each vehicle to the blockchain.

In order to include the variations of trust values in the blockchain as soon as possible, a PoS approach is jointly used with PoW. The sum of the absolute values of trust offsets is considered as the stake, and a block with a higher stake value is more likely to be elected. By using a joint PoS and PoW approach, Reference [58] was able to disseminate the timely update of the trust values while using PoW to prove the computing efforts. This approach has two main disadvantages. First, it is not a totally distributed approach as the creation of blocks depends on RSUs. Second, it only works in scenarios that have a sufficient number of RSUs, which is not applicable for most scenarios due to the cost of installing RSUs.

Vehicles need to improve their respective understanding about the environment based on notifications from other vehicles. Vehicles that detect a specific event can disseminate this information to other vehicles. However, upon reception of a message indicating a traffic event such as an accident warning, traffic congestion, and road construction information, the vehicle must first verify the validity of the information before taking the next action. However, this is challenging in decentralized vehicular networks. In [59], Yang et al. proposed a traffic message validation mechanism based on blockchain. The authors introduced a proof-of-event (PoE) consensus concept that evaluates each vehicle by collecting periodic messages that include vehicle heading, speed, and location information. The authors assumed that the RSUs have enough computational power and wired network connections with each other in order to create and maintain the blockchain.

Liu et al. [60] proposed a blockchain-based trust management scheme with the consideration of vehicle privacy. The scheme consists of two parts. The first part is an anonymous announcement and verification protocol. An initiator vehicle that receives a message indicating a traffic event invites other vehicles to verify the message. The verifiers reply with the corresponding signatures if they accept the invitation and successfully verify the signature of the initiator. Then, the initiator generates a new message by aggregating the signatures, and the message is verified by the nearest RSU. The second part is a blockchain-based trust management model. When an event happens, an RSU can receive multiple messages from different vehicles. The RSU first evaluates the reputation of each vehicle based on a logistic regression method and then determines the trust of the corresponding message. Then, the RSU works as a miner to create a block containing the reputation data to the blockchain.

Xie et al. [61] proposed a trust management approach for real-time video report in software-defined networking (SDN)-enabled VANETs. In [61], when a source vehicle uploads a video, the vehicle attaches the corresponding traffic condition tag, which is disseminated to other vehicles. The traffic tag information is evaluated by vehicles in the vicinity. After the reception of a video, an RSU evaluates the trustworthiness of the traffic information tag based on the evaluation results from the vehicles and the inter-vehicle distance (between the evaluator and the source vehicle). The RSU then creates a block and adds the block to the blockchain.

### 3.4. Networking Layer

There are some studies using blockchain to solve the problems of data communications in vehicular environments. In [62], the impact of vehicle mobility on the performance of blockchain was discussed by considering three metrics, namely the success probability of adding a block, the time period of rendezvous, and the maximum number of exchangeable blocks during a rendezvous. Most studies utilize the key advantages of blockchain, namely decentralization, irrevocability, and anonymity, to improve the performance of networking. We can classify these existing works according to the main problem that the blockchain solves, as shown in Table 3.

#### 3.4.1. Decentralization Purpose

Some studies utilize the decentralized feature of blockchain to design a system without depending on a centralized organization. Li et al. [63] employed a blockchain-based approach to replace the third-party service providers for data sharing in VANETs. By combining the ciphertext-based attribute encryption, Ethereum blockchain, and the interplanetary file system (IPFS) technologies, Reference [63] aimed to establish a distributed VANET data sharing platform with enhanced data security and privacy and a fine-grained access control scheme.

Zhang et al. [64] argued that the dynamic and infrastructure-less feature of VANETs could result in security risks, and proposed an SDN approach to manage VANETs. In order to solve the vulnerability of the conventional centralized control plane to malicious nodes, they proposed a block-based decentralized control plane to achieve a consensus among multiple controller nodes in complex vehicular IoT environments. The network architecture includes three different layers, namely the device layer, area control layer, and domain control layer. The device layer consists of vehicles, and the area control layer collects vehicle information and sends it to the domain control layer. A permissioned blockchain is used to achieve consensus among multiple domain controllers.

#### 3.4.2. Security Purpose

Zhang et al. [65] proposed a consortium blockchain-based data sharing framework for VANETs to solve the risk of malicious tampering in a centralized data storage scheme. The framework is as follows. First, each vehicle sends data to nearby RSUs. The RSUs work as preselected nodes and generate blocks according to the data received from vehicles. By achieving a consensus among RSUs, the blockchain that contains the vehicle data is generated. The smart contract technology is used to allow the RSUs to control the data sharing process. Based on the tamper-resistant feature of the blockchain, this data sharing framework aims to solve the security risks of the centralized data storage scheme.

Rawat et al. [66] integrated blockchain with named data networking to provide privacy-aware and secure data communications in distributed vehicular networks. The main contribution of [66] is that the maintenance of blockchain does not rely on RSUs or other fixed infrastructures. In [66], the vehicles are grouped into multiple clusters where each cluster may have multiple cluster heads. The cluster heads are the candidates for miners in the blockchain system. Specifically, for each cluster, one of the cluster heads is selected as a miner, and this miner can be replaced by another cluster head depending on the vehicle movements. Based on the consensus achieved among multiple cluster heads, Reference [66] is more attack-tolerant than the centralized approach as all data exchanges are verified by multiple cluster heads.

Wang et al. [67] considered the vulnerability of the centralized system to data tampering attacks targeting the central server and proposed a blockchain-enabled framework for secure content delivery in connected vehicle environments. A permissioned blockchain is used to speed up the consensus process. RSUs perform as full nodes (consensus nodes) to store the full ledgers while vehicles (ordinary nodes) only store the metadata. They introduced a proof-of-reputation (PoR) consensus protocol that is jointly used with the PoW approach to reach consensus. The reputation of a vehicle is evaluated by using a task-based approach based on cooperativeness, honestness, and the quality of task completion. The reputation of an RSU is calculated using a credit-based approach based on the behavior of the RSUs involved in the consensus process of the blockchain. Each authorized RSU can vote for a consensus node for ledger management whereby the voting weight is determined by the reputation value of the RSU. Before adding a block to the blockchain, a consensus node solves a PoW puzzle with a difficulty that is inversely proportional to the reputation value of the consensus node. By integrating the proof-of-reputation concept with PoW, Reference [67] can improve the speed of block generation at honest RSUs while adding more costs to attacks conducted by malicious RSUs.

In [68], Chen et al. employed a double-layer blockchain for secure data sharing in vehicular named data networks. The blockchain is used to avoid the spread of fake messages by maintaining a global consensus that includes negative and positive data sharing records. In order to improve the scalability of data sharing of named data networking, Reference [68] uses a double-layer blockchain approach. In the bottom layer, vehicles are divided into different groups based on their interests. Each group maintains a private blockchain where vehicles perform as miners to create blocks. In the top layer, multiples RSUs maintain a consortium blockchain to satisfy the need of data sharing among different vehicle groups. When a data request cannot be satisfied in the same group, the request is sent to nearby RSUs for a further search of matching resources.

Zhang et al. [69] proposed a lightweight permissioned blockchain-based approach to maintain the interest-key-content binding information for named data networking in unmanned aerial vehicle (UAV) ad hoc networks. The main purpose of [69] is to use blockchain to mitigate content poisoning by verifying the content name, publisher public key digest, and content digest. Adaptive delegate consensus algorithm (ADCA), which is a new consensus approach that does not require a mining procedure, is introduced to shorten the time required for reaching an agreement. ADCA consists of two processes, namely the normal process and the delegate consensus process. In the normal process, the ground control station verifies the blocks, resulting in the disadvantage of centralized control. In the delegate consensus process, some UAVs are selected as delegates, and all the delegates perform the verification tasks in a round-robin manner.

Shrestha et al. [70] discussed the security risks of a regional blockchain in VANETs in terms of the density of malicious vehicles, the complexity of the puzzle in PoW, and block message dissemination delay. They used computer simulations to show that the security risks can be basically eliminated if a certain condition is met. They derived the condition for security and provided a guideline for parameter selections in the design of a blockchain system.

#### 3.4.3. Incentive Purpose

Blockchain technology is also widely used to improve the incentive of collaboration in a decentralized network environment. Wang et al. [71] proposed a blockchain-based incentive scheme for vehicular energy networks (VENs) that facilitates the renewable energy trading over a large geographical area by using electric vehicles (EVs). They introduced a permissioned energy blockchain with a proof-of-reputation consensus protocol that reduces the block generation delay as compared with PoW. Some energy nodes (e.g., for energy storage) can be elected as validators, and they create blocks based on the proof-of-reputation protocol. The reputation of an energy node is defined according to the charging/discharging services provided. A pricing-based incentive approach is employed to optimize the charging/discharging activities of EVs for the purpose of reaching a balanced regional energy use.

In vehicular networks, after detecting an accident, a vehicle sends an announcement to other vehicles. In order to verify the trustworthiness of the announcement, the vehicle must cooperate with other vehicles (witnesses). However, sometimes, users lack the motivation to forward the announcement messages. In [72], Li et al. proposed CreditCoin, which is a blockchain-based incentive announcement network that motivates users to share traffic information. In the blockchain system, vehicles are users, and each vehicle is allocated a credit account to store reputation points (i.e., coins). RSUs or official public vehicles participate in the consensus phase of the blockchain and verify the blocks. By allocating coins for collaborative vehicles, the incentive for packet forwarding at each vehicle is improved significantly.

Wang et al. [73] proposed a blockchain-based rewarding scheme to incentivize battery-powered vehicles (BV) to contribute services for vehicle-to-grid networks. They classified the system entities into four different types, namely the central aggregator (CAG), local aggregator (LAG), BV, and blockchain. The CAG is an operator of the vehicle-to-grid network, which communicates with the electricity market on behalf of the BVs. The LAG is also a local operator with smaller size. Every local area has an LAG. The CAG authorizes the legal BVs to provide services and allocates rewards to the BVs through the blockchain. However, the problems of how to maintain the blockchain and achieve the consensus were not discussed in [73].

### 3.5. Application Layer

#### 3.5.1. Decentralization Purpose

The use of blockchain in designing decentralized systems is one of the most important research topics in the IoT era. Many applications achieve a consensus among participating network nodes. Current systems are typically dependent on a central server. Federated learning (FL) [6] is a distributed learning algorithm that receives increasing interests in the design of an intelligent systems with privacy protection. In FL, the central server aggregates feedback from multiple learning agents, namely local models, to establish the global model. Similar to FL, most distributed algorithms are not decentralized, and therefore, in order to design a decentralized system, blockchain technology is widely discussed. Table 4 and Table 5 show the existing research efforts on the application of blockchain in solving the application layer issues of vehicular IoT.

Pokhrel and Choi [74] proposed a blockchain-based approach to enable FL in a decentralized vehicular environment. The local models and different versions of the global models are maintained by the distributed ledger of blockchain, which is visible and verifiable by every vehicle. Vehicles work as miners to attend to the consensus process by verifying local updates from different workers (clients). A reward is given to a miner based on the size of local models aggregated from multiple clients, incentivizing clients to verify the blocks. By using the blockchain technology, Reference [74] is able to perform decentralized FL in vehicular environments. The effect of the block arrival rate on the performance of system is also discussed by simulation and numerical analysis.

Zhang et al. [75] used a consortium blockchain in traffic signal control with VANETs for the purpose of deploying intelligent transportation systems. They argued that a decentralized traffic signal control system is better than the centralized one in terms of resilience. They introduced a credit management mechanism to prevent vehicles from announcing fake messages. The traffic department allocates a positive reward to an honest vehicle that contributes to the system by sharing road condition information. A punishment is given to a malicious vehicle that provides fake information. RSUs work as miners and add blocks to the blockchain. By sharing the blocks among the networks nodes, the traffic signal is controlled for the purpose of reducing the average waiting time of vehicles. However, the time delay due to block verification and block announcement can have a significant impact on the traffic signal control efficiency, and this issue was not adequately discussed in [75].

Su et al. [76] proposed a decentralized data sharing approach for disaster rescue purposes using UAV-assisted vehicular networks. They used a blockchain to facilitate collaboration between UAVs and ground vehicles for secure and efficient data exchange in disaster areas. Two different types of transactions are defined in the blockchain. The first one is the transaction reporting the misbehavior of nodes. The other one is the transaction conducting normal data exchanges between nodes (including both UAVs and ground vehicles). A delegated proof-of-stake (DPoS) algorithm is used to reach consensus. All full nodes first vote for delegates, and the elected delegate nodes control the consensus process. Different from the conventional DPoS scheme, Reference [76] considers the credit of each node and the dissenting vote in the voting mechanism, which makes it more resilient to malicious nodes.

Shen et al. [77] discussed the privacy concern of support vector machine (SVM) training in vehicular social networks (VSN) and proposed a consortium blockchain-based system to decentralize the trailing process by avoiding data sharing with a third party. The system conducts most training operations at local service providers without sharing with others and aggregates the local training results based on the blockchain. This concept is similar to federated learning with the difference that [77] uses a blockchain to avoid aggregating data at the central server. The system architecture consists of three different layers, specifically the VSN device layer, VSN data provider, and blockchain service platform (BSP). The BSP allows VSN data providers to access all records saved on the blockchain and provides strong security by keeping the VSN data invisible to unauthorized entities.

In [78], Ma et al. employed blockchain to decentralize the key management process in VANETs. They used blockchain to enable a distributed storage for public keys and employed the smart contract technology to automate the registration and management of keys. Three types of entities are used in the key management process, namely vehicle service provider, blockchain network, and vehicles. The vehicle service provider deploys the blockchain network and defines the smart contract. The vehicle service provider also provides an interface to users by conducting identity management of vehicles, transaction data management, and public key management. The blockchain network is constructed by the RSUs that perform the functionalities of miners based on the PoW consensus algorithm. The blockchain technology is applied to speed up the public key update process by automating all the steps based on a blockchain and smart contract. The decentralized voting mechanism of the blockchain is also utilized to detect adversarial users.

Fu et al. [79] discussed the decentralization of network function virtualization management and orchestration (NFV-MANO) for IoV. They proposed a blockchain-enabled NFV framework to achieve coordination among multiple MANO systems without requiring a centralized control server. The throughput and delay of the blockchain are jointly considered in the framework design. The computational tasks of the blockchain are processed by using the edge computing technologies. The main focus of [79] lies in how to decentralize the NFV functionalities with blockchain technologies, and therefore, it does not seriously discuss the mobility issues of vehicles and their impact on the blockchain performance in the framework design.

Wang et al. [80] discussed the sharing of private parking slots in a decentralized manner based on the blockchain technology. Reference [80] protects user privacy by using anonymous credentials without a centralized third party credential allocator. The whole block network is maintained by multiple fog servers where each fog server is responsible for its own serving area by collecting and sharing parking slot information. Parking slot users pay parking fees through the blockchain network. The blockchain network verifies the payment transactions and records the verified transactions into the ledger. While [80] mainly discusses the decentralized parking slot sharing procedures, the details of how to achieve the consensus are not discussed.

With a similar purpose, Zhang et al. [81] proposed a blockchain-based decentralized parking system to achieve reliability and fairness in smart parking. The system includes five different types of entities, namely trusted authority, parking owner, driver, RSU, and the blockchain network. The trusted authority is in charge of generating public parameters and keys for each parking owner and driver. When a problem happens, the trusted authority is able to trace the problem source and retrieve the real-world identity of parking owners and drivers involved in the problem. A smart contract is used to achieve automation and fairness. If a driver pays the parking fee, it is guaranteed that the driver would obtain the corresponding parking slot. Similarly, a parking owner gets paid by providing private parking slots. The blockchain network consists of blockchain nodes that collaborate with each other to reach a decentralized consensus. However, the owner and distribution of blockchain nodes are not discussed.

In [82], Deng and Gao conducted a simple discussion about a blockchain-based payment system for VANETs. RSUs work as full nodes to maintain the blockchain, and vehicles generate the content of the transactions. The details of how to verify a block are not discussed in [82]. Hassija et al. [83] pointed out the importance of designing a lightweight blockchain for microtransactions and proposed an energy trading platform for vehicle-to-grid networks. They used the tangle data structure to store transactions by using the directed acyclic graph. They implemented the tip selection algorithm to allow adding new transactions without doing mining. A game theory model is also designed to determine prices in energy trading.

Yao et al. [84] employed a permissioned blockchain-based approach to enable a decentralized identity-as-a-service in vehicular cloud computing scenarios involving multiple distributed clouds. The system is composed of five entities, including the trusted authority, vehicular clouds, vehicles, vehicular cloud computing service providers, and a blockchain network. The trusted authority is responsible for publishing public parameters and ensuring the security of the whole system. All vehicles and service providers must be registered via the trusted authority. Vehicle clouds maintain the blockchain ledger. The main concern about this system is that the maintenance of the blockchain is highly dependent on the trusted authority.

Liu et al. [85] introduced an adaptive EV participation scheme based on blockchain technology to motivate EVs to participate in energy trading for the purpose of avoiding the significant fluctuation of power level and charging cost. They formulated the scheduling problem of EV charging and discharging and proposed a scheme based on the Icebergorder algorithm [114]. The participants of the system include the power plants, micro energy generators, energy storage, and consumers. All customers connect to a public blockchain where the electricity information and trading transactions are recorded. However, Reference [85] mainly proposes the scheduling algorithm and does not discuss the deployment and maintenance issues of blockchain.

In [86], Hassija et al. proposed a decentralized parking slot scheduling algorithm based on a directed acyclic graph (DAG). All users, including the parking slot owners and parking slot users, connect to the same distributed network. Since the order of parking slot allocation is important, the conventional PoW consensus approach is not applicable due to the possible risk of a fork occurring. In order to solve this problem, a DAG-based consensus algorithm is used to provide much faster transactions while avoiding any valid block from becoming a fork. The conventional PoW algorithm uses a large amount of computational power in the consensus. The Byzantine agreement approach is able to prevent the waste of computational power by taking votes from the users and owners. However, the conventional voting approach requires a large network bandwidth to send the votes, which affects the parking slot allocation efficiency and correctness. A virtual voting scheme that does not use extra bandwidth is proposed in [86]. The virtual vote is conducted based on a predefined algorithm without exchanging any voting information between voters.

Hassija et al. [87] proposed a decentralized energy trading framework between UAVs and charging stations based on a blockchain approach that uses DAG. By using the IOTAtangle [115] approach, Reference [87] eliminates the mining procedure of the conventional blockchain system and achieves equivalence among all the participants. The directed acyclic graph also ensures that the consensus can be achieved when most participants agree to a particular transaction. In [87], each user registers to the IOTA network and receives a unique ID and other information, including security keys and wallet addresses. UAVs are buyer nodes, and charging stations are seller nodes. UAVs can buy energy from the charging stations through the IOTA network with the exchange of IOTA tokens. If a buyer does not have enough tokens for a transaction, the buyer can first borrow some tokens from the corresponding charging stations and then repay the charging stations later with interest. If the buyer is not able to repay the borrowed tokens in a predefined time, late fees are charged. These communications and transactions between buyers and sellers are executed by a smart contract, ensuring the validity of the system.

#### 3.5.2. Security or Privacy Purpose

Gabay et al. [88] discussed the possible privacy leakage problem for EVs in the charging process and proposed a privacy-aware authentication problem based on blockchain and zero-knowledge proofs. The Ethereum distributed ledger is used to achieve a decentralized consensus, and a zero-knowledge proof-based approach is adopted to enable privacy protection. The approach of zero-knowledge proofs allows an EV to verify its own charging behavior without revealing its identity. The entities in the system include EVs, the EV service provider, and the blockchain network. First, the EV service provider generates a secret function using the information of the EV and passes the function with a proving key to an EV. The EV then uses the secret function to generate a witness and combines it with the proving key to create a proof. The EV contacts a smart contract of the blockchain network where the proof is used to validate the EV. The smart contract verifies the proof and passes a service token to the EV. The service token is used in charging without disclosing any information of the EV.

Iqbal et al. [89] proposed a blockchain-based decentralized trust management scheme in VANETs for the purpose of evaluating whether a fog vehicle is suitable for offloading tasks or not. They discussed a scenario where RSUs offload some tasks to fog vehicles in the vicinity and discussed a fog vehicle selection algorithm that takes into account the workload and reputation of vehicles. A consortium blockchain is employed to maintain the reputation of vehicles where RSUs act as miners. A proof-of-elapsed-time (PoET) approach is used to achieve consensus among multiple RSUs. In PoET, a random waiting time is scheduled for each miner, and the miner that has the shortest waiting time is elected to add new blocks to the blockchain. The blockchain maintains two ledgers, namely the transaction ledger and reputation ledger. The transaction ledger records all the successful offloading tasks including the vehicle identifiers, meta information, offloading requests, computational demands, task deadlines, and information of selected fog vehicles. The reputation ledger records the social reputation scores of vehicles for task offloading that are considered in the task offloading node selection.

A remote attestation model for trusted computing in vehicular environments based on blockchain was introduced in [90]. The attestation process involves two steps. The first step is the identity authentication, which verifies whether a node has a legal identifier or not. The second step includes making decisions and adding blocks to the blockchain. The blockchain of [90] maintains a decentralized ledger of access control decisions, whereby each participating nodes is identified by an attestation identity key. Experiments are conducted considering a real V2X scenario to validate the model.

Huang et al. [91] leveraged the decentralization and tamper-proof audit feature of blockchain to achieve better security in vehicular fog computing. They used a blockchain with a smart contract to enable decentralized computation offloading. A large-scale application of the blockchain technology in parked vehicle assisted fog computing is discussed, and a Stackelberg game-based approach is introduced to optimize the design of the smart contract. The related entities in the blockchain system include requesters, performers, and miners. A requester is a network node that has some tasks to offload to other entities. The performers include fog servers and the parked vehicles that can help the requester to conduct task executions. RSUs and fog servers act as miners based on PoW. The smart contract works as follows. A requester submits a task offloading request to the blockchain, and the blockchain assigns an agent to process the request. The offloading request is visible to all performers. After some performers indicate positive responses about the task offloading, the agent selects task executer nodes among the performers and calculates the rewards for the selected performers. The requester agrees to the reward calculated by the agent by making a deposit. Tasks are offloaded to the executers, and the output results are written to the smart contract, while a special transaction is triggered to authorize miners to verify the output results. If some results do not satisfy the requirements, backup performers are activated automatically to support task executions. The agent aggregates results from multiple task executers and sends the final result to the requester. Finally, the requester pays the task offloading fees to the agent, and the performers contributing to the task offloading are paid accordingly.

In [92], Liang et al. pointed out the problem of existing intrusion detection systems (IDS), specifically being deployed on predefined static strategies, and proposed a two-layer blockchain scheme to intrusion detection systems for vehicular networks. Specifically, for each local area, a micro-blockchain is used to maintain the local samples and detection strategies specialized for the local environments. A control plane is designed to automatically improve the IDS based on local intrusion information collected by the micro-blockchain. Multiple micro-blockchains then construct a macro-blockchain to cover different types of samples and strategies. The macro-blockchain is able to handle spatial-temporal dynamics using global knowledge to support local vehicles. Since each micro-blockchain is responsible for fast collection and analysis of the data in the corresponding area, a practical Byzantine fault tolerance (PBFT)-based lightweight consensus mechanism is used. The performance of the scheme is evaluated by comparing it with the conventional IDS.

Li et al. [93] discussed an advertisement dissemination scenario through V2X communications that allows service providers to promote their products. In order to motivate vehicles to forward the advertisement, the security and privacy issue must be considered. For example, some malicious vehicles possibly try to defraud the advertiser to get a reward without contributing to the dissemination of the advertisement, which is called the “free-riding” attack. It is important to design a fair and efficient protocol that can verify the dissemination behaviors of vehicles with low storage and communication cost. Meanwhile, possible risks about privacy leakage also discourage the advertising behaviors of vehicles. To solve these problems, Li et al. proposed a blockchain-based scheme that combines the Merkle hash tree with smart contracts to mitigate the “free-riding” attack. A zero-knowledge proof technology is employed to achieve anonymity and protect privacy. The scheme considers four different entities, namely register authority, advertisers, vehicles, and RSUs. The register authority is in charge of the key generation and identity management. Vehicles act as forwarders of advertisement and receive the corresponding reward for the advertisement dissemination. RSUs act as consensus nodes to validate transactions and maintain the blockchain. The advertisers send advertisement requests to nearby RSUs and provide advertisement fees for the purposes of attracting sufficient vehicles in the process of the advertisement.

Wang et al. [94] proposed an identity authentication scheme for IoV based on blockchain. They argued that the blockchain technology can be integrated with the public key infrastructure to achieve an efficient identity authentication and avoid misuse of vehicle accounts in IoV. They used Ripple’s consensus algorithm in which the information about the voting nodes are disclosed to users. However, the determination of the voting nodes was not seriously discussed in [94].

In [95], Lu et al. also mentioned that the privacy-preserving authentication is the first priority issue for defending against attacks in VANETs and proposed a blockchain-based solution. They pointed out that the conventional centralized trusted authority (TA)-based solution has two main problems. First, the acclivities are not visible by the vehicles, which raises some security risks. Second, a receiver must check a certificate revocation list (CRL) for the authentication of a message, which incurs a high computational overhead. To solve these problems, Reference [95] employs a permissioned blockchain approach to use multiple semi-trusted authorities in a decentralized manner. A Merkle Patricia tree is used to provide a cryptographically authenticated data structure. The conventional CRL-based verification of a certificate is replaced by a hash function-based approach that shows a much shorter verification latency. Each vehicle is assigned multiple certificates for the purpose of privacy protection. The relationship between a certificate and the corresponding real identity is encrypted and recorded in the blockchain. The authentication scheme is implemented on the Hyperledger Fabric (HLF) platform and evaluated by comparing with other baseline approaches.

Feng et al. [96] proposed a consortium blockchain-assisted authentication scheme to verify the credibility of messages transmitted in VANETs. The privacy issue is also considered in the authentication scheme. The scheme consists of five modules, namely the initialization module, smart contract deployment module, vehicle registration module, login and authentication module, and vehicle revocation module. The initialization module generates the system parameters and initializes the consortium blockchain. The trusted authority selects some blockchain manager nodes and uses the PBFT consensus mechanism to maintain the blockchain. The smart contract module adds the functions of smart contracts to the blockchain. Each vehicle is securely registered by the vehicle registration module. The login and authentication module controls the authentication process of message transmissions in vehicular networks. The vehicle revocation module is in charge of updating the status of a vehicle in smart contracts when the vehicle state changes due to vehicle movement or other reasons. The scheme is implemented on the Hyperledger Fabric platform.

Zheng et al. [97] proposed a blockchain-based secure authentication scheme for V2X communication to prevent transmissions of forged messages and preserve the privacy of vehicles. Two types of transactions, namely the transactions recoding vehicle identities and transactions containing traffic announcement, are considered in the scheme. Distributed cloud storages are integrated with the blockchain to maintain the vehicle identity-related transactions, and therefore, the size of transactions written to the blockchain is reduced while protecting the privacy and integrity of transaction contents. The hash value of the traffic announcement-related transactions is recorded in the blockchain. Depending on some cloud storages, the scheme is not totally decentralized.

In [98], Sheikh et al. used blockchain technology to secure energy trading in EV networks. They used a Byzantine-based consensus algorithm to achieve consensus in a decentralized environment. The energy trading process is formulated using the Byzantine Generals problem by considering different types of attacks, and a decentralized solution based on blockchain is discussed. The solution is evaluated using a well-used IEEE test feeder, and the security advantage over the conventional approach is discussed in [98].

Li et al. [99] discussed the secure distribution of group keys for UAV ad hoc networks and proposed a solution based on blockchain. The ground control station (GCS) establishes a private blockchain to manage group keys. The authors also integrated a mutual-healing approach with the blockchain to improve the key distribution process in a lossy and unreliable environment. The sender adds some redundancies to data messages including group keys, which ensures that the group members can recover some lost group keys. A group member also can recover some lost messages by requesting assistance from its neighbors. In [99], a private blockchain is used, and the GCS is the only entity that generates blocks. In order to improve the consensus process in an unreliable environment where packet losses and long transmission delays exist, Reference [99] employs a mutual-healing method to recover lost blocks by retrieving some data from their neighbors. The authors conducted computer simulations to show that the integration of the mutual-healing scheme with the blockchain technology is more resistant to various attacks as compared with existing mutual-healing schemes.

Yao et al. [100] considered cross-data center authentication in vehicular fog services (VFS) and argued that the conventional cross-data center authentication approach over geographically distributed regions does not seriously address the privacy issue and delay requirement of driving vehicles. They proposed a blockchain-based authentication mechanism for distributed VFS, which has the following features. First, based on the blockchain technology, the mechanism provides anonymity and resistance to attacks by solving the single point of failure problem. Second, by combining the cryptographical and blockchain technologies, the mechanism is able to reduce the size of data exchanged between a vehicle and a service manager in the authentication process. A vehicle only needs to transmit a request message before accessing a vehicular fog service. The size of the communication between different service managers is also reduced in the authentication process.

Dorri et al. [101] proposed a public blockchain-based architecture to improve the security and privacy level of vehicular IoT systems. The architecture uses an overlay network consisting of overlay nodes (i.e., overlay block managers) that manage the blockchain. The overlay nodes can be vehicles, original equipment manufacturers, service providers, and mobile devices. Several use cases including the remote software updates, insurance, EV charging services, and car sharing services are discussed to explain the importance of the architecture. Focusing on the architecture design, some details of the consensus mechanism are not addressed.

Huang et al. [102] proposed a blockchain-based scheme for secure charging pile management in EV charging and discharging applications. They defined a decentralized security model by using the lightning network and smart contract technology in the blockchain system. The scheme includes four phases, namely the registration phase, scheduling phase, authentication phase, and charging phase. EVs, charging piles, and operators are registered in the registration phase. Four different scheduling algorithms, specifically the shortest path first, minimum time cost first, minimum comprehensive cost first, and minimum waiting time first scheduling, are considered in the charging piles’ allocations. A two-way authorization approach is used between an EV and a charging pile. In the charging phase, the charging process is completed, and the transactions are written into the blockchain. While [102] mainly discusses the operational process of charging pile management, it does not discuss the details of blockchain management.

In [103], Tan and Chung used a consortium blockchain to manage the authentication and group key distribution in VANETs. The group membership records are maintained in the blockchain, which improves the group management process. The blockchain also maintains the identities of participating vehicles in the key updating process. The authentication scheme includes two phases, namely the offline registration phase and the authentication phase. The offline registration phase is managed by the trusted authority, and all the participating entities must register for the system. A dynamic group management approach is designed based on the Chinese remainder theorem. The system model uses a hierarchical architecture that includes the cloud layer, edge layer, and user layer. The maintenance of the blockchain is not clearly discussed in [103].

Guo et al. [104] proposed a blockchain-based authentication scheme for vehicular IoT. The scheme uses a hierarchical architecture consisting of the vehicular network layer, blockchain edge layer, and blockchain network layer. The vehicular network layer provides an interface and services to the vehicles, and the blockchain network layer maintains the blockchain. The nodes contributing to the blockchain consensus are the network nodes on the Internet, and the selection of the blockchain network nodes is not discussed in [104]. An authentication mechanism based on the integration of the blockchain technology and a digital signature algorithm enables trusted authentication. Each vehicle can upload its information and the corresponding privacy policy to the blockchain network through an interface provided by the blockchain edge network. The blockchain network creates blocks to store the information of the vehicles. The blockchain network also uses smart contracts to automate the authentication process. When a moving vehicle enters a new area, the digital signature algorithm is used to verify the vehicle.

Wang et al. [105] proposed a blockchain-based trustworthiness computation scheme for VANETs. The scheme involves three different types of entities, namely vehicles, RSUs, and the blockchain network. The blockchain network records the characteristics and the trustworthiness of the vehicles. RSUs collect vehicle information and calculate the trustworthiness value of each vehicle, which is recorded in the blockchain. RSUs are also responsible for conducting the authentication process. For a handover between two RSUs, an efficient handover authentication approach is also designed. When a vehicle moves from the coverage of an RSU to another RSU, the previous RSU sends a handover certificate to the next RSU and the vehicle. Since all the trustworthiness information of the vehicle is shared in advance by the blockchain, the latter RSU only needs to check whether the trustworthiness information is still valid or not.

Gao et al. [106] introduced a combination of blockchain and SDN technology for VANETs in the 5G and fog computing era. The practical Byzantine fault tolerance (PBFT) consensus algorithm is used to ensure consistency among multiple entities involved in the system. RSUs serve as miners, and a leader is elected among them to create a block. Some preselected nodes participate in the voting process to verify a block before a consensus is reached. Every node in a public blockchain can also vote if they can solve a PoW puzzle. Each RSU can access the whole transaction records of the blockchain. By using the immutable feature of the blockchain, the trustworthiness of a message can be evaluated, and then, the reputation score for each vehicle is recorded in the blockchain. The SDN technology is used to achieve the optimization of resource allocation, mobility management, and policy management. However, instead of using the SDN controller to fully control the network, the functionalities are shared among RSUs using the blockchain technology.

In [107], a blockchain-based privacy-aware content distribution mechanism in vehicular environments was discussed by Qian et al. The authors argued that the conventional way of distributing content upon requests faces some privacy issues as it reveals some information of requesters. To solve this problem, a content acquisition approach based on broadcast communications is discussed. RSUs can disseminate contents using broadcast communications, and a vehicle can receive contents whenever necessary. If a vehicle starts to obtain contents, a content transaction occurs. The blockchain records each content transaction, and all the transactions are broadcast to all content providers for the purpose of authentication and audit. While [107] discusses the framework of the content distribution, some important issues still need to be further investigated. First, since the throughput of broadcast communications is much smaller than the unicast communications, the data distribution performance of the system can be reconsidered. Second, it is difficult to know whether a vehicle receives a broadcast message or not as there is no acknowledgment for a broadcast frame. Third, the consensus algorithm of the blockchain also needs to be carefully designed in order to satisfy the data dissemination requirements in dynamic vehicular environments.

As discussed above, some studies use blockchain technologies to improve the privacy of vehicular IoT systems. In these studies, the privacy is mainly achieved without disclosing the identities of users in the physical world. However, all transactions are known by all the blockchain users. This also increases the possibility of privacy leakage since some private information of the users can be deduced from analyzing the big data of blockchain transactions.

#### 3.5.3. Audit Purpose

Some studies focus on the exploration of the audit functionality of the blockchain technology. Abbade et al. [108] used a blockchain to record the vehicle odometer data for the purpose of dealing with the odometer fraud, which may result in dangerous accidents. By utilizing the audit feature of blockchain technology, they proposed a blockchain architecture to register the odometer data of vehicles and share them in an open and decentralized manner. The system consists of three blocks, namely the data insertion block, the blockchain network, and data visualization block. The data insertion block provides an interface for vehicles to send the incremental difference of odometer data to the blockchain network. The blocks are created by master nodes and verified by all the nodes in the network. A block is accepted by the blockchain network if it collects $\frac{2}{3}$ positive votes, which follows the practical Byzantine fault tolerance proposition.

In [109], Kong et al. introduced a permissioned blockchain-based immutable data sharing scheme for vehicular fog computing. The scheme involves three different types of entities, namely vehicles, RSUs, and server. The server is responsible for distributing keys and initial parameters to RSUs and vehicles. RSUs collect vehicle sensor information and create transactions that record the vehicle sensory data. A small group of selected RSUs acts as miners and generates blocks. A new block is broadcast to the entire network and verified by other RSUs and the server. The block is approved if it receives sufficient positive votes that satisfy the Byzantine fault tolerance consensus algorithm. All the entities are able to read the transactions included in the blockchain. This enables a transparent and immutable data sharing platform for vehicles.

Singh et al. [110] proposed a blockchain-based data integrity management scheme for data processing in V2X environments. The scheme uses a layered architecture consisting of five layers, namely the V2X layer, task creation layer, container management layer, edge layer, and blockchain management layer. The first layer accepts the data processing requests from vehicles. The task creation layer creates tasks according to the data processing requests and forwards the tasks to the container management layer, which assigns containers to the tasks. The edge layer assigns user requests to the suitable containers at edge nodes based on the container-to-task mapping suggested by the container management layer. The blockchain management layer records data from different sources to the block. The details of the miner selection and the consensus algorithm are not discussed in [110].

Lu et al. [111] used the blockchain technology to assist federated learning for data sharing in vehicular environments. They considered a vehicular scenario that involves three types of entities, including vehicles, RSUs, and macro base stations. RSUs act as MEC servers. A privacy-aware knowledge sharing mechanism is achieved by conducting local training at each vehicle and then aggregating local models. This is beneficial for all the vehicles. However, the knowledge exchange between vehicles and RSUs must be strictly monitored for the purpose of ensuring the performance of federated learning. Blockchain is used to verify the qualities of exchanged local model parameters in order to enhance the reliability of the global model in the federated learning process. A delegated PoS-based consortium blockchain is used. Some RSUs are voted as delegates by vehicles and manage the blockchain including the control of the block intervals and block sizes. The historical performance of RSUs is also considered in the delegation scheme.

Cebe et al. [112] utilized the immutable feature of blockchain to support forensics applications in vehicular IoT. They employed a permissioned blockchain-based framework to record vehicle-related data to the blockchain, which can be used to detect the reasons after an accident based on the vehicle sensor data. The framework involves the following parties: vehicles, vehicle maintenance service providers, vehicle manufacturers, law enforcement, and insurance companies. Vehicular sensor data, including the data from the event data recorders (EDRs) and controller area network data, are periodically recorded to the blockchain. Since the blockchain data are accessible by all the parties, they can be used as proofs for post-accident scenarios. The blockchain network is misaligned by three different types of nodes, namely leader nodes, validator nodes, and monitor nodes. The validator nodes are the nodes owned by the manufacturers, maintenance centers, and insurance companies. Some validator nodes are elected as leader nodes, and the PBFT approach is used to reach consensus. The monitor nodes are owned by the law enforcement authorities and do not participate in the consensus process, but keep all the records for the purpose of post-disaster scenarios.

With a similar purpose of auditing the exchange of distributed learning models in federated learning, Reference [113] introduces a blockchain-based collective learning framework for connected vehicles. The framework consists of three different parts, namely users, MEC nodes, and a blockchain system. Vehicles are the users that want to utilize the knowledge of other vehicles through the collective learning. RSUs or base stations serve as edge nodes to collect the local training models from vehicles and aggregate the collected and verified models. The learning models are added to the blockchain by the MEC nodes. A delegated PoS consensus approach is considered to satisfy the Byzantine fault tolerance. However, the details of the delegation algorithm are not addressed in [113].

## 4. Discussions

Table 6 shows the main characteristics of existing blockchain studies. Here, “SC” stands for “smart contract” and “Pub.” publication. We use “–” to denote that the corresponding information is not provided in the paper. “Mgmt. entity” stands for “management entity,” which shows the entity generating the blocks where “server” denotes a server (cloud server or edge server) having wired connection with the cloud. The network architecture and the block generation throughput are shown by “N/W architecture” and “throughput,” respectively.

From Table 6, we can observe that some studies discuss the use of smart contract, while some do not. There is a diversity in the consensus algorithms, while DAG has been achieving great interest in recent years due to its high block generation throughput as compared with other consensus algorithms. Most studies use RSUs, cloud servers, or edge servers as miners. A small number of studies discuss the use of vehicles as miners [66,68,70,74,76,83,86,90]. The current studies basically assume that the vehicles are connected to the blockchain network through the RSUs. A few studies [66,76,86,87] discuss the blockchain management in distributed networks. Regarding the evaluation approach, most authors use computer simulations or theoretical analysis to evaluate their proposals, while only one paper [102] uses real-world experiments. The performance of the block generation throughput and the efficiency of achieving consensus are not discussed adequately in all the existing studies.

We summarize the limitations of the existing studies as follows.

- Most studies only discuss the importance of using blockchain in some applications and do not seriously address the details of the blockchain maintenance and corresponding impact on the system performance. Since computer simulations cannot reveal all the problems, more investigations should be conducted based on the real-world implementation of blockchain systems.
- The use of blockchain in a distributed network is not discussed adequately. Most studies consider the use of RSU or cloud servers as the miners to generate blocks, which is not applicable for distributed environments such as vehicular ad hoc networks.
- Blockchain management in a wireless environment is a very challenging topic, which is under-explored in the literature. In order to achieve a decentralized agreement, blockchain systems need to broadcast the transaction records to all the miners. This requires a large communication bandwidth, which is quite difficult to satisfy in a resource limited vehicular environment.
- The mobility impact on the blockchain performance is not discussed adequately. The problem of “how to reach a consensus in a mobile environment” should be discussed.

## 5. Future Research Directions

In order to achieve the high reliability and ultra-low latency required by most vehicular IoT applications, it has become particularly important to efficiently utilize the computing, communication, and computing resources in a decentralized manner. This has brought about many opportunities for blockchain technologies and has opened up new research topics as follows.

### 5.1. How to Design an Efficient Blockchain System to Satisfy the Application Requirements in Vehicular IoT?

- Blockchain framework for emerging vehicular IoT applications: Different vehicular IoT applications have different features and possibly different QoS requirements. For example, the collision avoidance systems requires an ultra-low latency, while vehicle data analytics systems have stricter requirements on the computing. It is important to design an efficient blockchain framework considering the characteristics of each application. More research efforts are required to define efficient blockchain frameworks for emerging vehicular IoT applications.
- Privacy-aware blockchain: The dissemination of all transaction histories in the whole network forms the basis of achieving consensus in a blockchain system. This incurs a privacy concern because all transaction details are disclosed to authorized nodes, resulting in the possibility of revealing the real identity of an account. Especially in the big data era, the combination of data from different entities can increase the possibility of privacy leakage. Therefore, there is an urgent need to discuss “how to protect the privacy of all users participating in blockchain transactions” by considering the features of vehicular IoT environments.
- Security issues: Although the security level of blockchain is higher than the conventional centralized system, there is also a concern of the 51 percent attack [54]. Due to the vehicle mobility, some legitimate vehicles may fail to contribute to the blockchain system on time, causing a security problem. Therefore, the problem of “how to avoid security attacks in a dynamic network environment” needs further investigations.
- Blockchain for collaborative intelligence: A large number of devices, vehicles, sensing devices, communication devices, and computing devices are present in vehicular IoT. Due to the limited communication and computing resources at each vehicle, future vehicular IoT can consider collaboration between multiple entities to achieve a higher computational capability and lower latency. In recent years, federated learning [5] has attracted great interest in utilizing the knowledge from multiple devices to improve the intelligence of a system. The conventional federated learning relies on a centralized server to aggregate feedback from different clients. However, in some vehicular IoT environments, the centralized control server does not exist. Blockchain facilitates collaboration among multiple entities without relying on any centralized server. The problem of how to use blockchain to improve the intelligence of a decentralized system with collaborative intelligence can be an interesting research topic.

### 5.2. How to Improve Vehicular IoT Protocols to Support Blockchain

- Network performance improvement for blockchain: In a blockchain system, a large volume of data is exchanged in the network before achieving global consensus. Most studies have yet to address the large amount of networking overhead incurred in maintaining a blockchain, which is especially a significant issue in vehicular networks where network bandwidth is limited. While blockchain provides a decentralized approach, it incurs much higher overhead than the centralized approach due to the need to disseminate the transaction details to the whole network. Therefore, it is important to consider the tradeoff between decentralization and networking overhead. The study of the networking performance of blockchain in vehicular IoT environments is an urgent and under-explored topic. More effort can be made to improve the networking performance of blockchain in vehicular environments by combining with the networking [116] and computing technologies [117], as well as decentralized consensus algorithms.
- Communication, computing, and caching strategies for blockchain: The performance of a blockchain system is affected by the joint allocation of communication, computing, and storage resources. Since there is no centralized control server in a blockchain system, joint resource allocation becomes more complex. Vehicular IoT environments exhibit new challenges to the resource allocation strategies due to the dynamic network topology. First, the limited communication resource can be a bottleneck in enabling a decentralized system. Second, some computation jobs can be conducted by a collaborative approach because each single vehicle may not have enough computational capability to support a computation-intensive job. Third, conducting some efficient caching of data at some nodes can be beneficial for improving the whole system performance. The studies on the communication, computing, and caching strategies are expected to attain great research interest in the future.
- Vehicular environment-aware blockchain: The design of a blockchain system must cater to the characteristics of vehicular environments, including varying vehicle mobility, vehicle densities, multi-access communication environments, and the diversity of applications. For example, some existing studies use RSUs as miners to add blocks to the blockchain. Since frequent handovers may occur in vehicle-to-roadside (V2R) communication scenarios, vehicles connecting to the same RSU change over time. The vehicle density also varies with time and location. All these features must be considered in the design of a consensus algorithm. Different communication interfaces can be integrated with edge computing technologies to compensate the limitation of each communication technology [118]. There is also a need to adjust the tradeoff between reliability and latency in designing a consensus algorithm as some applications require an ultra-low latency [119].

## 6. Conclusions

Blockchain has been attracting increasing interest from both academic and industrial sectors due to its promising features of providing a decentralized, open, and immutable system. Many new interesting functions and applications are possible when blockchain is employed in vehicular IoT environments. Meanwhile, the dynamicity of vehicular environments poses great challenges to the design of an efficient blockchain system.

Existing studies discuss the use of blockchain technologies in solving the problem of different IoT layers, namely perception, networking, and application layers. For the perception layer, blockchain is mainly used to solve the trust management issue in decentralized environments or improve the perception accuracy by sharing data among the vehicles. For the networking layer, blockchain is mainly used to solve decentralized networking, network security, and incentivization for networking. A large amount of studies focus on the application layer issues with different purposes of decentralization, security/privacy, and audit. Since different applications involve different types of network entities and require different levels of QoS, the design of an efficient blockchain system targeting a specific scenario is still an open problem. Future vehicular IoT systems require an efficient integrated design of different IoT layers. For example, an efficient perception about a complex environment requires the collaboration among multiple vehicles, where blockchain can be used to achieve a consensus among different vehicles. Therefore, there is still room for research on the use of blockchain in vehicular IoT systems.

On the other hand, with the emergence of communication and computing technologies, it becomes important to discuss the efficient use of communication and computing resources for the purpose of supporting blockchain in vehicular environments. Vehicular environment-aware blockchain protocols should be designed to satisfy the ultra-reliable low latency requirements of vehicular IoT applications. The issues of guaranteeing the performance of blockchain in dynamic vehicular environments require further explorations.

In this paper, we first explain the fundamentals of blockchain and then explain the recent efforts on the application of blockchain in vehicular IoT. After discussing existing solutions and open technical issues, we point out future research directions regarding the combination of blockchain and vehicular IoT. We discuss both directions of “how to improve vehicular IoT protocols to support blockchain” and “how to design an efficient blockchain system to satisfy the application requirements in vehicular IoT”. We believe this study can expedite the research activities of both blockchain and vehicular IoT technologies and facilitate the deployment of blockchain technologies in vehicular IoT applications.

## Figures and Tables

**Table 1:** Comparison of different types of blockchain.

	Public Chain	Consortium Chain	Private Chain
Participant	Anyone	Consortium member	Individual/single organization
Consensus mechanism	PoW/PoS/DPoS	Distributed consensus algorithm	Distributed consensus algorithm
Recorder	All participants	Based on consortium member agreement	Customized
Incentive mechanism	Necessary	Optional	Not necessary
Features	Trust establishment	Efficiency and cost optimization	Transparency and traceability
Throughput	3–200,000 tps	1000–10,000 tps	1000–100,000 tps

**Table 2:** Blockchain for perception layer issues in vehicular IoT.

Purpose	Publication	Research Summary
Positioning	Li et al. [57]	A blockchain-based technology to store and share the evolution of positioning errors in order to protect the security of cooperative vehicles.
Trust management	Yang et al. [58]	A blockchain-based decentralized trust management system where RSUs work as miners to create blocks; a combination of PoS and PoW is used in achieving consensus.
	Yang et al. [59]	A traffic message validation mechanism, which is based on blockchain, that uses a PoE consensus concept.
	Liu et al. [60]	A blockchain-based trust management scheme with the consideration of vehicle privacy.
	Xie et al. [61]	A trust management approach for real-time video report in SDN-enabled VANETs.

**Table 3:** Blockchain for networking layer issues in vehicular IoT.

Purpose	Publication	Research Summary
Decentralization	Li et al. [63]	A data sharing approach that combines the ciphertext-based attribute encryption, Ethereum blockchain, and the interplanetary file system technologies, in VANETs.
	Zhang et al. [64]	A permissioned blockchain-based approach for SDN in VANETs.
Security	Zhang et al. [65]	A consortium blockchain-based scheme for VANETs to solve the risk of malicious tampering.
	Rawat et al. [66]	An integration of blockchain technology and named data networking to provide privacy-aware and secure data communication in distributed vehicular networks.
	Wang et al. [67]	A permissioned blockchain-enabled framework for secure content delivery in connected vehicle environments.
	Chen et al. [68]	A double-layer blockchain for secure data sharing in vehicular named data networks. Multiples RSUs maintain a consortium blockchain to satisfy the need of data sharing among different vehicle groups.
	Zhang et al. [69]	A blockchain-based approach to avoid content poisoning by verifying the content name, publisher public key digest, and content digest.
	Shrestha et al. [70]	A discussion on the security risks of a regional blockchain in VANETs.
Incentive	Wang et al. [71]	A blockchain-based incentive scheme for vehicular energy networks.
	Li et al. [72]	A blockchain-based incentive announcement network called CreditCoin that improves the motivation of users in sharing traffic information.
	Wang et al. [73]	A blockchain-based rewarding scheme to improve the incentive of battery-powered vehicles for contributing services in vehicle-to-grid networks.

**Table 4:** Blockchain for application layer issues in vehicular IoT (Part 1).

Purpose	Publication	Research Summary
Decentralization	Pokhrel and Choi [74]	A blockchain-based approach to enable federated learning in a decentralized vehicular environment.
	Zhang et al. [75]	A consortium blockchain-based traffic signal control scheme for VANETs toward intelligent transportation systems.
	Su et al. [76]	A decentralized data sharing scheme for disaster rescue purposes in UAV-assisted vehicular networks.
	Shen et al. [77]	A consortium blockchain-based decentralized system for VSNs.
	Ma et al. [78]	A blockchain-based approach to decentralize the key management process in VANETs.
	Fu et al. [79]	A blockchain-based approach for achieving the decentralization of the NFV functionalities.
	Wang et al. [80]	A blockchain-based private parking slot sharing scheme.
	Zhang et al. [81]	A blockchain-based parking system to achieve reliability and fairness in smart parking.
	Deng and Gao [82]	A blockchain-based payment system for VANETs.
	Hassija et al. [83]	A lightweight blockchain for energy trading platform in vehicle-to-grid networks.
	Yao et al. [84]	A permissioned blockchain-based approach for identity-as-a-service in vehicular cloud computing scenarios involving multiple distributed clouds.
	Liu et al. [85]	An adaptive EV participation scheme based on blockchain to motivate EVs to participate in energy trading for the purpose of balancing energy usage and costs.
	Hassija et al. [86]	A decentralized parking slot scheduling algorithm based on DAG.
	Hassija et al. [87]	A decentralized energy trading framework between UAVs and charging stations based on DAG.
Security/Privacy	Gabay et al. [88]	A privacy-aware authentication scheme based on blockchain and zero-knowledge proofs for EVs in the charging process.
	Iqbal et al. [89]	A blockchain-based decentralized trust management scheme for task offloading in VANETs.
	Xu et al. [90]	A remote attestation model for trusted computing in vehicular environments.
	Huang et al. [91]	A blockchain to achieve better securities in vehicular fog computing.
	Liang et al [92]	A two-layer blockchain scheme for intrusion detection systems in vehicular networks.
	Li et al. [93]	A blockchain-based scheme that combines the Merkle hash tree with smart contracts to mitigate the “free-riding” attacks in an advertisement dissemination scenario through V2X communications.
	Wang et al. [94]	A blockchain-based identity authentication scheme for IoV.
	Lu et al. [95]	A blockchain-based solution for privacy-preserving authentication in VANETs.
	Feng et al. [96]	A consortium blockchain-assisted authentication scheme for message verification in VANETs.
	Zheng et al. [97]	A blockchain-based secure authentication scheme for V2X communication to eliminate forged messages and preserve the privacy of vehicles.

**Table 5:** Blockchain for application layer issues in vehicular IoT (Part 2).

Purpose	Publication	Research Summary
Security/Privacy	Sheikh et al. [98]	A blockchain-based security management scheme for energy trading in EV networks.
	Li et al. [99]	A blockchain-based approach for secure distribution of group keys for UAV ad hoc networks.
	Yao et al. [100]	A blockchain-based authentication mechanism for distributed vehicular fog services.
	Dorri et al. [101]	A public blockchain-based architecture to improve the security and privacy level of vehicular IoT systems.
	Huang [102]	A blockchain-based scheme for secure charging pile management in EV charging and discharging applications.
	Tan and Chung [103]	A consortium blockchain to manage the authentication and group key distribution in VANETs.
	Guo et al. [104]	A blockchain-based authentication scheme for vehicular IoT where a smart contract is used to automate the authentication process.
	Wang et al. [105]	A blockchain-based trust computation scheme for VANETs.
	Gao et al. [106]	A combination of blockchain with SDN for VANETs where the PBFT consensus algorithm is used to ensure consistency among multiple entities involved in the system.
	Qian et al. [107]	A blockchain-based privacy-aware content distribution framework in vehicular environments.
Audit	Abbade et al. [108]	A blockchain-based audit scheme for dealing with odometer fraud.
	Kong et al. [109]	A permissioned blockchain-based immutable data sharing scheme for vehicular fog computing.
	Singh et al. [110]	A blockchain-based data integrity management scheme for data processing in V2X environments.
	Lu et al. [111]	A blockchain to assist federated learning for data sharing in vehicular environments.
	Cebe et al. [112]	A blockchain to support forensics applications in vehicular IoT.
	Fu et al. [113]	A blockchain-based collective learning framework for connected vehicles.

**Table 6:** Main characteristics of existing blockchain studies. Pub., publication; SC, smart contract; Mgmt., management; N/W, network.

IoT layer	Pub.	SC	Consensus	Mgmt. Entity	Evaluation	N/W Architecture	Throughput
Perception	[57]	✓	DPoS	RSU	Simulation	RSU-based	Moderate
	[58]	–	PoS with PoW	RSU	Simulation	RSU-based	Moderate
	[59]	–	PoE	RSU	Simulation	RSU-based	Moderate
	[60]	–	PoW with PBFT	RSU	Simulation	RSU-based	Moderate
	[61]	–	PoS with PoW	RSU	Simulation	RSU-based	Moderate
Networking	[63]	✓	–	RSU	Simulation	RSU-based	Moderate
	[64]	✓	DPoS	Server	Simulation	Server-based	Moderate
	[65]	✓	PoW with PBFT	RSU	Theoretical	RSU-based	Moderate
	[66]	–	–	Vehicle	Simulation	Distributed	Moderate
	[67]	–	PoR with PoW	RSU	Simulation	RSU-based	Moderate
	[68]	✓	PoW	RSU and vehicle	Simulation	Hierarchical	Moderate
	[69]	–	Self-designed	GCS and UAV	Simulation	Hierarchical	Moderate
	[70]	–	PoW	Vehicle	Simulation	RSU-based	Moderate
	[71]	–	PoR	Charging station	Simulation	Infrastructure-based	Moderate
	[72]	–	PBFT	RSU	Simulation	RSU-based	Moderate
	[73]	✓	–	RSU	Simulation	Infrastructure-based	Moderate
Application	[74]	–	PoW	RSU or Vehicle	Simulation	Infrastructure-based	Moderate
	[75]	✓	BFT	RSU	Theoretical	RSU-based	Moderate
	[76]	–	DPoS	UAV or Vehicle	Simulation	Distributed	Moderate
	[77]	✓	-	Server	Simulation	Infrastructure-based	Moderate
	[78]	✓	PoW	RSU	Simulation	RSU-based	Moderate
	[79]	–	BFT	Server	Simulation	Infrastructure-based	Moderate
	[80]	–	–	Server	Simulation	Infrastructure-based	Moderate
	[81]	✓	–	Server	Simulation	RSU-based	Moderate
	[82]	✓	–	RSU	Simulation	RSU-based	Moderate
	[83]	✓	DAG	Vehicle or server	Simulation	Infrastructure-based	Good
	[84]	–	Self-designed	Server	Simulation	Infrastructure-based	Moderate
	[85]	✓	–	Server	Simulation	Infrastructure-based	Moderate
	[86]	✓	DAG	Vehicle or others	Simulation	Distributed	Good
	[87]	✓	DAG	UAV or others	Simulation	Distributed	Good
	[88]	✓	–	Server	Simulation	Infrastructure-based	Moderate
	[89]	–	PoET	RSU	Simulation	RSU-based	Moderate
	[90]	–	–	Vehicle or others	Simulation	Infrastructure-based	Moderate
	[91]	✓	PoW	RSU or server	Simulation	Infrastructure-based	Moderate
	[92]	–	BFT and PoW	Server	Simulation	Hierarchical	Moderate
	[93]	✓	–	RSU	Simulation	RSU-based	Moderate
	[94]	✓	BFT	Server	Simulation	RSU-based	Moderate
	[95]	–	PoP	RSU	Simulation	RSU-based	Moderate
	[96]	✓	PBFT	Server	Simulation	RSU-based	Moderate
	[97]	–	–	RSU	Simulation	RSU-based	Moderate
	[98]	✓	BFT	–	Simulation	Infrastructure-based	Moderate
	[99]	–	Self-designed	GCS	Simulation	Infrastructure-based	Moderate
	[100]	–	PBFT	Server	Simulation	Infrastructure-based	Moderate
	[101]	✓	PoW	Server	Simulation	Infrastructure-based	Moderate
	[102]	✓	–	Charging station	Experiment	Infrastructure-based	Moderate
	[103]	–	–	–	Simulation	Infrastructure-based	Moderate
	[104]	✓	–	Server	Simulation	Infrastructure-based	Moderate
	[105]	–	–	RSU	Simulation	RSU-based	Moderate
	[106]	✓	PBFT	RSU	Simulation	RSU-based	Moderate
	[107]	–	–	Server	Simulation	RSU-based	Moderate
	[108]	✓	PBFT	Server	Simulation	Infrastructure-based	Moderate
	[109]	–	BFT	RSU	Simulation	RSU-based	Moderate
	[110]	–	–	Server	Simulation	Infrastructure-based	Moderate
	[111]	–	DPoS	RSU	Simulation	RSU-based	Moderate
	[112]	–	PBFT	Server	Simulation	Infrastructure-based	Moderate
	[113]	–	DPoS	RSU	Simulation	RSU-based	Moderate

## Abbreviations

The following abbreviations are used in this manuscript:

BFT	Byzantine fault tolerance
CRL	Certificate revocation list
DAG	Directed acyclic graph
DLT	Distributed ledger technology
DPoS	Delegated proof-of-stake
DTN	Delay tolerant network
EV	Electric vehicle
FL	Federated learning
GCS	Ground control station
GPS	Global positioning system
IoT	Internet of Things
IoV	Internet of Vehicles
ITS	Intelligent transport systems
MEC	Mobile edge computing
ML	Machine learning
NFV	Network functions virtualization
PBFT	Practical Byzantine fault tolerance
PoE	Proof-of-event
PoET	Proof-of-elapsed-time
PoP	Proof-of-presence
PoR	Proof-of-reputation
PoS	Proof-of-stake
PoW	Proof-of-work
QoS	Quality of service
RSU	Road side unit
SDN	Software-defined networking
UAV	Unmanned aerial vehicle
URLLC	Ultra-reliable low-latency communications
V2R	Vehicle-to-roadside
V2V	Vehicle-to-vehicle
V2X	Vehicle-to-everything
VANET	Vehicular ad hoc networks
VSN	Vehicular social networks
